# Tissue perfusion and its influencing factors in epigastrial adipocutanous flaps affected by ischemia-reperfusion in rats

**DOI:** 10.1590/acb410226

**Published:** 2026-01-16

**Authors:** Anna Orsolya Flasko, Laszlo Adam Fazekas, Gergo Kincses, Adam Varga, Adam Attila Matrai, Lili Dorottya Csoka, Sandor Zahorszki, Anna Toth, Csaba Filler, Tamas Juhasz, Abel Molnar, Norbert Nemeth

**Affiliations:** 1 University of Debrecen – Faculty of Medicine – Department of Otorhinolaryngology and Head and Neck Surgery – Debrecen – Hungary.; 2 University of Debrecen – Doctoral School of Clinical Medicine – Debrecen – Hungary.; 3 University of Debrecen – Faculty of Medicine – Department of Operative Techniques and Surgical Research – Debrecen – Hungary.; 4 University of Debrecen – Faculty of Medicine – Department of Surgery – Debrecen – Hungary.; 5 University of Debrecen – Faculty of Medicine – Department of Anatomy, Histology and Embriology – Debrecen – Hungary.; 6 University of Debrecen – Faculty of Medicine – Department of Dermatology – Debrecen – Hungary.

**Keywords:** Ischemia, Reperfusion, Blood Circulation, Hemorheology, Microcirculation, Tensile Strength, Histology

## Abstract

**Purpose:**

In reconstructive surgery, complications of flaps include thrombosis and necrosis, partly originated from ischemia-reperfusion (I/R) injury. Therefore, investigations on the factors that influence tissue perfusion are essential. We wished to investigate microcirculation, micro-rheological factors, histomorphological, and biomechanical alterations of adipocutaneous flaps with/without I/R.

**Methods:**

In anesthetized rats, groin flaps were prepared bilaterally. On the left side, the vascular pedicle was clamped for 2 hours before re-suturing the flaps. Skin temperature and microcirculation were monitored before/after surgery and on the first, third, seventh, and 14th postoperative days, besides blood samplings for testing hematological parameters, erythrocyte deformability and aggregation. At the end of experiment, skin samples were taken for histological and tensile strength examinations.

**Results:**

The hematological and micro-rheological parameters reflected the acute phase reactions, showing erythrocyte deformability impairment and enhanced aggregation. The microcirculatory values of the ischemic flaps were lower than the contralateral ones even two weeks after surgery. The ischemic-side flaps shrank to a greater extent. Histology revealed that mastocyte number decreased, and the quantity and organization of collagen fibers were altered in ischemic flaps.

**Conclusion:**

The microcirculatory and micro-rheological alterations during the regeneration of the flaps were well observed. Flap ischemia modulated the tissue perfusion parameters, tensile strength, collagen content, and fiber organization.

## Introduction

The outcome and the success of reconstructive surgical procedures using various flaps strongly depends on the appropriate perfusion of the flaps^1^^–^ 5. The tissue perfusion is determined by the morphological and functional status of the vasculature (including microcirculation), the hemodynamics, as well as the macro- and micro-rheological parameters^6^^,^ 7. For monitoring the perfusion, as viability of the flaps, several methods are known that can be used in the clinical practice and in experimental studies as well^8^^–^ 16. Complications of flap surgery include thrombotic events, ischemia-reperfusion injury, flap necrosis, originating from torquation, distortion of the supplying vessels, comorbidities, and inflammation^5^^,^ 7 ^,^ 17 ^–^ 19.

Numerous animal models are used for studying these issues, performing flaps of various tissue composition (fasciocutaneous, adipocutaneous, musculocutaneous, etc.) and with different ischemic times^20^^–^ 25. In previous studies, we have investigated microcirculatory and hemorheological relations of fascio-, adipocutaneous, and musculocutaneous flaps in large and small animal models, revealing that early microcirculatory and micro-rheological deterioration can be predictive for postoperative complications^26^^–^ 29. However, numerous questions are still unanswered related to the tissue composition, localization, and duration of ischemic time. It is not completely known how pedicle blood flow, hemorheological factors, and tissue microcirculation are related to each other.

In this study, the aim was to simultaneously follow-up the alterations of factors affecting tissue perfusion on standardized adipocutaneous groin flaps with or without ischemia-reperfusion injury.

## Methods

### Experimental animals

The experiment has been registered and officially approved by the University of Debrecen’s Committee of Animal Welfare and the National Food Chain Safety Office (registration number 19/2022/UDCAW), in accordance with the national law (Act XXVIII of 1998 on the Protection and Humane Treatment of Animals) and European Union regulations (Directive 2010/63/EU).

Ten male Wistar rats (bodyweight: 362.7 ± 17.08 g, origin: Toxi-Coop Zrt., Budapest, Hungary) were included in this pilot study. The rationale for using adult male rats were providing homogeneity of sex and the appropriate size for the experimental surgical interventions. The animals were kept in the department’s conventional animal facility. General anesthesia was induced using 100 mg/bwkg ketamine + 10 mg/bwkg xylazine intraperitoneally^30^^,^ 31.

### Operative techniques and sampling protocol

**Figure 1:**
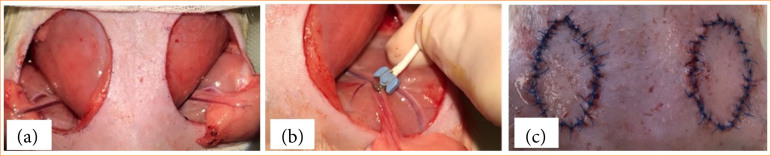
Intraoperative photos of **(a)** bilateral groin adipocutaneous flap preparation, **(b)** blood flow measurement of flap vascular pedicle (superficial epigastric artery), and **(c)** the re-sutured flaps.

Source: Elaborated by the authors.

After shaving and disinfecting the skin, on the epigastrial region, standard adipocutaneous flaps were prepared bilaterally (surface: 400.76 ± 66.08 mm^2^) pedicled on the superficial epigastric artery and vein (Fig. 1). On the left side, the flap pedicle was clamped for 2 hours, while the flaps were placed in the wound beds. After the ischemic period, the microvascular clamp was removed, the flaps were repositioned and sutured with 32 tension-free interrupted stitches (4/0 Pidilen, Kollsut, United States of America) on both sides, and we treated the wounds with 0.6-mL hydroxypropyl-methylcellulose (HPMC) gel. The daily wound gel treatment was kept until the fifth postoperative (p.o.) day (0.15 mL/flap/day). On the first three p.o. days, 15-mg/kg/day tramadol was administered intraperitoneally^32^. In the p.o., the animals were kept individually, and had a plastic collar to prevent autophagy. Daily wound care was done. Wound debridement and/or resuturing were needed in two cases. We performed these procedures under general anaesthesia.

Photographs were taken of the flaps after surgery, and on the first, third, seventh, and 14^th^ p.o. days, to calculate the size of the flaps (ImageJ 1.40 g freeware).

Skin surface temperature was also measured (Rodent NIBP infrared thermometer with LaserSight, AD Instruments, United States of America) on both flaps and on intact epigastrial skin region (middle region) before operation, during ischemia, after operation (after re-suturing the flaps), and on the seventh p.o. day.

Blood samples were taken from the lateral tail vein before operation and on the first, third, seventh, and 14th p.o. days. On the last day, the animals were euthanized by an overdose of anesthetics, and skin samples (standard size: 0.5 × 3 cm) were excised from the flap’s upper, lateral, and lower regions (including flap part – suture line – intact skin part), and from intact abdominal skin areas for tensile strength tests and histological examinations.

### Blood flow test of flap pedicle

We assessed blood flow in the epigastric superficial artery using the Transonic T206 (Transonic Systems, Ithaca, United States of America) device on both sides before and after the ischemic period (before re-suturing) (Fig. 1b) and on the 14^th^ p.o. day by gentle preparation of the flap vascular pedicle. Positioning the device’s probe directly on the pedicle vessel provided real-time flow measurements in mL/min^33^^,^ 34.

### Microcirculatory investigation

A Cytocam-IDF (Braedius Medical B.V., Huizen, Netherlands) videomicroscopy device was used to assess microcirculation by epi-illuminating superficial tissue layers (depth: 2–3 mm) with incident light, allowing hemoglobin-containing red blood cells to appear as black or gray dots against a bright tissue background^35^^,^ 36.

The microcirculatory recordings were offline analysed expressing perfused vessel density (PVD [mm/mm^2^]), proportion of perfused vessels (PPV [%]), and microvascular flow index (MFI [au])^35^^–^ 37.

### Laboratory methods testing hematological and micro-rheological parameters

A Sysmex K-4500 automated system (TOA Medicor Electronics Co., Ltd., Japan) was used to assess hematological parameters. In this study, white blood cell count (WBC [10^9^/L]), red blood cell count (RBC [10^12^/L]), hematocrit (Hct [%]), hemoglobin concentration (Hgb [g/dL]), mean corpuscular volume (MCV [fL]), mean corpuscular hemoglobin content (MCH [pg]), mean corpuscular hemoglobin concentration (MCHC [g/dL]), and platelet count (Plt [10^9^/L]) were analyzed.

A LoRRca MaxSis Osmoscan ektacytometer (Mechatronics BV, The Netherlands) was used to test RBC deformability^38^^,^ 39, determining elongation index (EI) in the function of shear stress (SS [Pa], range = 0.3–30 Pa) in samples of 10-µL anticoagulated blood and 2 mL of polyvinyl-pyrrolidone solution (PVP in phosphate-buffered saline, viscosity = 33.3–33.6 mPas, osmolarity = 290–310 mOsm/kg, pH = 7.2). All measurements were carried out at 37°C^39^. Individual EI-SS curves were compared using the EI values at 3 Pa, the maximal elongation index (EImax), the shear stress at half EI_max_ (SS_1/2_, [Pa]), and their ratio (EI_max/_SS_1/2_), calculated by the Lineweaver-Burk equation^40^.

A Myrenne MA-1 aggregometer (Myrenne GmbH, Germany) was used to test RBC aggregation determining M index values under stasis (M 5 s, M 10 s) and M1 values at 3 s^-1^ shear rate (M1 5 s, M1 10 s)^38^. The measurements were taken at room temperature (20–25°C). Each index parameter was determined using four parallel measurements, the average of which we used.

### Tensile strength measurements

We used a tensile strength testing device developed in collaboration with the Department of Information Technology^41^^,^ 42. The excised samples (5 × 20 mm) were secured between the clamping jaws at a distance of 8 mm, ensuring that the suture line was centered. The pulling force generated by the motor (1.95 mm/s) was recorded in grams, and the data was exported to a CSV file.

For data processing, the force values were converted from grams to newtons (9.81 m/s^2^), and both the maximum force (breaking point) and the slope of the force-time curves were analyzed^41^^,^ 42.

### Histological analysis

The area of interest on the skin was excised and removed on the 14^th^ p.o. day. The skin samples were fixed onto dental wax and subsequently placed in formalin for two days. The use of dental wax was essential to prevent the samples from shrinking and curling. After fixation, the samples were washed three times with distilled water, followed by an ascending series of alcohol, and then embedded in paraffin. Serial sections of 7 µm of thickness were prepared using a microtome (Leica, Wetzlar, Germany).

The samples were stained with dimethyl-methylene blue (DMMB) dissolved in water (Sigma-Aldrich, St. Louis, MO, United States of America), hematoxylin-eosin (H&E, Sigma-Aldrich, MO, USA), and picrosirius red (Sigma-Aldrich, MO, United States of America), following the manufacturer’s guidelines. The slides were then covered with DPX (Sigma-Aldrich, MO, United States of America). Histological slides stained with DMMB and H&E were examined under a light microscope (BX53 Olympus, Tokyo, Japan) with consistent camera settings and exposure.

For the samples stained with picrosirius red, observations were made using a polarized light, *i.e.*, the plane of polarized light was rotated by λ/4 and analyzed with a λ/4 compensator using the BX53 Olympus microscope (Olympus, Tokyo, Japan). Photographs were taken in both normal and polarized light under fixed camera settings. Following picrosirius red staining of the tissue samples, we measured the thickness of collagen fibers with detection of red fibers representing thick collagen fibers and green fibers representing thin collagen fibers. We used ImageJ 1.40 g software for red and green pixel analysis. After DMMB staining, mast cells were counted in the area of interest in 10× magnification slides, and the total cell count was recorded.

### Statistical analysis

To determine the sample size (number of animals per group), the Mead’s resource equation method was used. Data were presented as means ± standard deviation (S.D.). Statistical analyses were performed by a SigmaStat Software 3.1.1.0 (Systat Software Inc., San Jose, CA, United States of America). After normality test, one-way and repeated-measure analysis of variance (ANOVA) or Kruskal–Wallis’ test were used for intra-group comparison. The significance level was set for *p* < 0.05.

## Results

### General observations, flap size, and skin temperature

**Figure 2:**
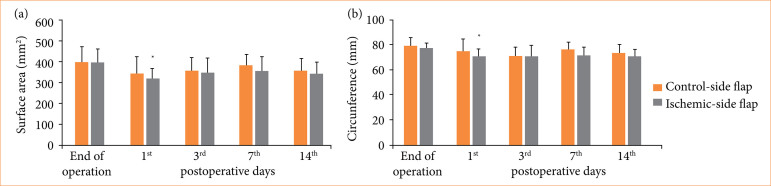
Changes of control-side and ischemic-side **(a)** flaps’ surface area (mm^2^) and **(b)** circumference (mm) during the observation period. Means ± standard deviation.

There were no thrombotic complications or visible flap necrosis during the observation period. The size of the flaps (surface area and circumference) decreased, and the ischemic-side flaps shrank to a greater extent (on the first day area: *p* = 0.007; circumference: *p* = 0.024) (Fig. 2).

*p < 0.05 versus base. Source: Elaborated by the authors.

Skin temperature decreased during the ischemic time, as flaps were prepared and left in a collapsed position for 2 hours. After re-suturing, the values were normalized and did not show notable alterations (Table 1).

**Table 1:** Changes skin surface temperature (°C) on intact epigastrial skin surface and on the control-side and ischemic-side flaps before, during, and after the ischemia, and on the seventh and 14^th^ postoperative days*.

Region	Before ischemia (base)	During ischemia	After ischemia	Seventh postoperative day	14^th^ postoperative day
Intact skin	36.00 ± 0.64	35.61 ± 1.17	36.38 ± 0.63	37.10 ± 0.95	37.36 ± 0.35
Control-side flap	35.75 ± 0.63	34.13 ± 1.34	35.70 ± 0.59	36.11 ± 1.39	36.62 ± 0.33
Ischemic-side flap	35.73 ± 0.56	33.92 ± 0.9	35.63 ± 0.85	36.40 ± 1.23	36.70 ± 0.23

* Means ± standard deviation.

Source: Elaborated by the authors.

### Hematological parameters

Table 2 shows selected quantitative and qualitative hematological parameters. WBC slightly decreased by the first p.o. day (*p* = 0.022 *versus* base), and started to be elevated over the observation period (14^th^ day: *p* = 0.017 *versus* base). RBC, hemoglobin, and hematocrit values decreased during the first postoperative week (seventh day: *p* = 0.003, *p* = 0.007 and *p* = 0.003 *versus* base, respectively), and by the 14^th^ day they tended to normalize. MCV, MCH, and MCHC values did not show important changes. Platelet count increased by the third (*p* = 0.027 versus base) and by the 14^th^ p.o. day (*p* < 0.001 *versus* base).

**Table 2:** Alterations of white blood cell count (WBC), red blood cell count (RBC), hemoglobin concentration (Hgb), hematocrit (Hct), mean corpuscular volume (MCV), mean corpuscular hemoglobin (MCH), mean corpuscular hemoglobin concentration (MCHC), and platelet count (Plt)*.

Variable	Base	First p.o. day	Third p.o. day	Seventh p.o. day	14^th^ p.o. day
WBC [×10^9^/L]	10.61 ± 3.76	7.32 ± 2.84**	10.78 ± 4.04	14.18 ± 7.43	13.62 ± 2.32**
RBC [×10^12^/L]	8.10 ± 0.61	7.84 ± 0.67	7.76 ± 0.35	6.85 ± 1.16**	7.55 ± 0.77
Hgb [g/dL]	15.07 ± 0.64	14.81 ± 1.29	14.61 ± 0.82	13.01 ± 2.39	14.23 ± 0.89
Hct [%]	46.61 ± 3.51	45.1 ± 4.19	44.23 ± 2.59	38.95 ± 7.49**	44.55 ± 4.71
MCV [fL]	57.24 ± 2.73	57.51 ± 2.31	56.98 ± 2.26	56.57 ± 2.4	57.43 ± 1.95
MCH [pg]	18.19 ± 1.94	18.92 ± 0.95	18.83 ± 0.81	18.95 ± 0.67	18.9 ± 0.97
MCHC [g/dL]	32.41 ± 1.19	32.85 ± 0.95	33.05 ± 0.65	33.48 ± 0.76	32.92 ± 0.72
Plt [×10^9^/L]	685 ± 142.45	728.33 ± 95.36	829.08 ± 154.47**	589.91 ± 301.39	952 ± 164.96**

* Means ± standard deviation;

** *p* < 0.05 versus base; p.o.: postoperative.

Source: Elaborated by the authors.

### Red blood cell deformability and aggregation

Elongation index at 3 Pa decreased by the seventh p.o. day (*p* = 0.003 versus base) and normalized at the end of the observation period. The EImax values moderately decreased mostly on the first and third p.o. days and normalized by the 14th day. SS1/2 values did not change significantly, but it slightly increased by the seventh and 14^th^ days (Table 3).

**Table 3:** Changes of elongation index (EI) at 3 Pa, maximal EI (EI_max_), shear stress at half EI_max_ (SS_1/2_), their ratio, and aggregation index (M 5 s, M1 5 s, M 10 s, M1 10 s) values.

Variable	Base	First p.o. day	Third p.o. day	Seventh p.o. day	14^th^ p.o. day
EI at 3 Pa	0.340 ± 0.032	0.342 ± 0.022	0.339 ± 0.018	0.317 ± 0.025**	0.330 ± 0.011
EImax	0.565 ± 0.018	0.546 ± 0.039	0.534 ± 0.036	0.553 ± 0.024	0.567 ± 0.017
SS_1/2_ [Pa]	1.99 ± 0.42	1.77 ± 0.36	1.96 ± 0.35	2.16 ± 0.32	2.13 ± 0.34
EI_max_ / SS_1/2_ [P^a-1^]	0.297 ± 0.068	0.320 ± 0.074	0.280 ± 0.052	0.261 ± 0.038	0.272 ± 0.045
M 5 s	2.23 ± 1.31	5.36 ± 2.22**	2.54 ± 0.86	1.69 ± 0.77	2.70 ± 1.67
M1 5 s	2.60 ± 1.31	4.94 ± 2.45**	3.01 ± 1.26	1.75 ± 0.73**	2.24 ± 1.21
M 10 s	5.50 ± 3.78	8.55 ± 4.50**	5.18 ± 2.78	3.68 ± 1.36**	6.75 ± 3.53
M1 10 s	5.55 ± 3.82	8.78 ± 4.55**	6.33 ± 4.16	4.28 ± 0.86	8.74 ± 5.07**

*Means ± standard deviation;

** *p* < 0.05 versus base; p.o.: postoperative.

Source: Elaborated by the authors.

Notable changes were seen in the red blood cell aggregation index values. By the first p.o. day, all the four index values increased (M 5 s: *p* < 0.001, M1 5 s: *p* < 0.001, M 10 s: *p* = 0.005, M1 10 s: *p* = 0.014 *versus* base) and remained elevated, then showed a rise again by the end of the observation period (M1 10 s: *p* = 0.022 *versus* base) (Table 3).

### Flap pedicles’ blood flow and microcirculatory alterations

The arterial blood flow values in the flaps’ pedicles were lower in the ischemic side (0.77 ± 0.24 mL/min) compared to the control side (0.90 ± 0.26 mL/min) just after the ischemic period. By the 14^th^ p.o. day, further decrease was seen, markedly in the previously ischemic flaps (control-side flap: 0.64 ± 0.25 mL/min; ischemic-side flap: 0.60 ± 0.17 mL/min). However, the changes were not significant.

**Figure 3:**
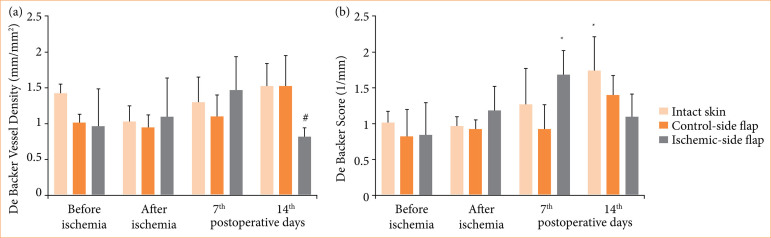
Alterations of microcirculatory score values, as **(a)** De Backer vessel density and **(b)** de Backer score, tested on intact abdominal skin part, on control-side and ischemic-side flaps, before and after ischemia and on the seventh and 14th postoperative days. Means ± standard deviation.

After flap preparation, De Backer vessel density decreased compared to intact skin. By the seventh p.o. day, perfusion values increased in the ischemic-side flap, and dropped by the 14^th^ p.o. day. Microcirculatory score values showed an elevation by the first week in the ischemic flaps, then values dropped at the end of the observation period (Fig. 3).

*p < 0.05 versus base; # versus intact skin and control-side flap. Source: Elaborated by the authors.

### Tensile strength

The tensile strength values and the slope data of force – strain curves are summarized in Table 4. Compared to intact skin, the tensile strength values were significantly lower at the suture line of the flaps in all flap parts and directions (*p* < 0.001). In horizontal pulling direction, the tensile strength values were higher compared to vertical direction (intact skin tensile strength: *p* = 0.008, curve slope: *p* = 0.005).

**Table 4:** Tensile strength values and slope of force – strain curves of tissue samples taken on the 14^th^ p.o. day from the upper, lateral, and lower parts of the flaps, and from intact skin part*.

Tissue sample	Region/direction	Tensile strength [N]	Slope of force – strain curve
Intact skin	Vertical	13.73 ± 5.48	0.062 ± 0.022
	Horizontal	31.57 ± 11.49 #	0.167 ± 0.062 #
Control-side flap	Upper (vertical)	3.39 ± 2.07**	0.024 ± 0.016**
	Lateral (horizontal)	2.77 ± 1.46**	0.030 ± 0.016**
	Lower (vertical)	2.12 ± 0.86**	0.018 ± 0.004**
Ischemic-side flap	Upper (vertical)	3.09 ± 1.38**	0.021 ± 0.016**
	Lateral (horizontal)	3.17 ± 1.53**	0.032 ± 0.007**
	Lower (vertical)	2.31 ± 0.55**	0.025 ± 0.012**

* Means ± standard deviation;

** *p* < 0.05 versus intact skin (identical direction); # versus vertical.

Source: Elaborated by the authors.

### Histology

**Figure 4:**
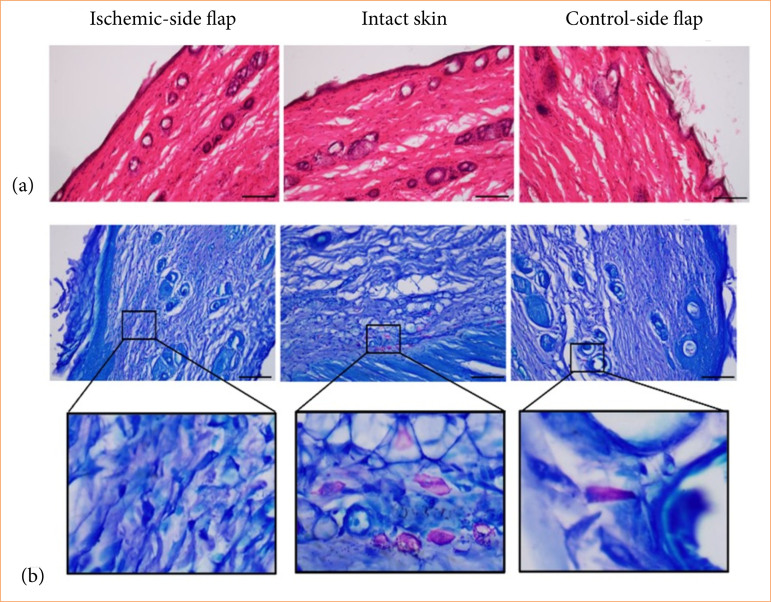
Representative histological photos of **(a)** hematoxylin-eosin and **(b)** dimethyl-methylene blue stained sections of ischemic-side flaps, intact skin, and control-side flaps. Biopsies were taken on the 14^th^ postoperative day. Original magnification: 20×, scalebar = 100 µm.

Source: Elaborated by the authors.

Histological analysis utilizing H&E staining has elucidated the inflammatory and healing processes in ischemic flaps. In both the ischemic and control skin samples, as well as in the contralateral side, no significant morphological changes were observed. The thickness of the epithelium and the granulation tissue did not demonstrate any pathological abnormalities. Additionally, the integrity of the dermis remained unaltered (Fig. 4a).

With dimethil-methylene blue staining, the metachromatically stained mast cells can be visualized. The quantity of mastocytes was reduced in the ischemic flaps compared to the intact skin and to the contralateral control side (Fig. 4b, Table 5).

**Table 5:** Changes on the number of mast cells and collagen fiber thickness (red: thick fibers, green: thin fibers) in intact skin part, in the control-side and ischemic-side flaps*.

Tissue sample	Number of mastocytes per 0.5 mm^2^	Collagen fiber thickness [pixel intensity]
Intact skin	23.25 ± 5.1	red: 25.3 ± 5.4
		green: 5.4 ± 1.3
Control-side flap	18 ± 4.4**	red: 34.7 ± 4.4**
		green: 7.6 ± 1.6
Ischemic-side flap	10 ± 6** #	red: 17.8 ± 6.4** #
		green: 5.8 ± 2.9

* Means ± standard deviation;

** *p* < 0.05 versus intact skin;

# *p* < 0.05 control side flap.

Source: Elaborated by the authors.

**Figure 5:**
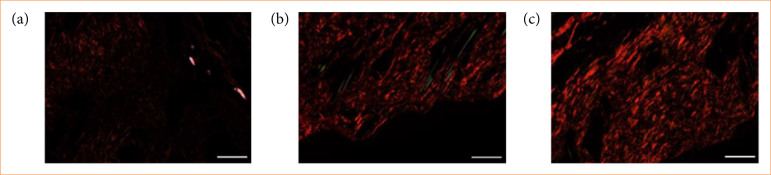
Representative histological photos of picrosirius red staining followed by rotation of polarized light with λ/4 of **(a)** ischemic-side flaps, **(b)** intact skin, and **(c)** control-side flaps. Biopsies were taken on the 14th postoperative day. Red color: thick fibers; green color: thin fibers. Original magnification: 20×, scalebar = 100 µm.

Source: Elaborated by the authors.

In picrosirius red stained sections, collagen is visualized, with non-specific staining appearing in red (thick fibers) and in green (thin fibers) color. When the plane of polarized light is rotated by λ/4, the birefringent structures reveal the organization of collagen fibers: thicker fibers appear as shiny red, while thinner fibers manifest as green. Our analysis showed that both red (thick fibers) and green (thin fibers) light intensities significantly decreased in the ischemic side compared to the intact skin and contralateral side. Notably, the red light intensity exhibited a significant decrease (*p* = 0.004) when compared to contralateral control side (Fig. 5, Table 5).

## Discussion

Reconstructive surgical procedures using various flaps of different localization, tissue compositions (*e.g.*, fasciocutaneous, adipocutaneous, musculocutaneous, etc.), vascular supply, and type (*e.g.*, local, rotated, transferred, interpolated, etc.) are commonly used, but numerous challenges are known^1^^–^ 5 ^,^ 43. Choosing the right kind of flap is essential, especially in case of larger defects, in which flaps of multiple components are preferred. Also, preserving the proper blood flow of the flap is crucial to maintain its viability. That requires well thought-out preoperative planning, precise operative technique, and proper postoperative care. Flap failure usually occurs when mistakes are made in the aforementioned stages of the reconstructive process. Monitoring flap viability is essential for the surgeon as noticing a decrease in it makes it possible to save the flap from necrosis.

Numerous techniques are described, which can be useful for assessing the vitality of the flap^8^^,^ 13 ^,^ 44 ^–^ 50. Although there are many possibilities to choose from, there is still no gold standard technique. The more advanced the reconstructive technique (*i.e.*, large flap size, free flap or buried flap), the more important the proper monitoring technique is.

In our study, we aimed to investigate several approaches for the assessment of the flap vitality including direct ultrasound examination of the pedicle, videomicroscopy, temperature measurements, hematological and micro-rheological parameters. Alterations in blood cell counts (red blood cells, leukocytes, platelets), hemoglobin and hematocrit, as well as changes in red blood cell deformability and aggregation, reflected the acute phase reactions after surgery during the inflammatory and early granulation phase of the wound healing^18^^,^ 19 ^,^ 51. It has also been demonstrated in the literature that these alterations occur in the early postoperative days after ischemia-reperfusion of flaps^11^^,^ 23 ^,^ 26 ^–^ 29. As red blood cell deformability and aggregation are influenced by changes in oxygenation level, acid-base parameters, free radical reactions, inflammatory processes^7^^,^ 26 ^,^ 29, they altered accordingly: red blood cell deformability impaired, erythrocyte aggregation enhanced in the early postoperative days. Impaired micro-rheology may contribute to tissue perfusion deterioration^6^^,^ 7. We could observe related alterations in the videomicroscopy recordings.

Concerning the changes in tensile strength, it has to be emphasized that the follow-up period of the study was limited. We could not investigate the entire wound healing process. In clinical care, stitches are usually removed on the seventh–14^th^ p.o. days, and the end of our study was also designed to be on the 14^th^ p.o. day. Our focus was on the early postoperative period. However, we could see early differences in the regeneration in comparision of intact skin, non-ischemic and ischemic flaps. The orientation and organization of the collagen fibers showed differences: in ischemic flaps, the wound healing and related regeneration processes were disturbed.

As the flaps, after their preparation, positioning and suturing, behave differently compared to intact skin, we have investigated the tensile strength at various parts of the flap. These values can be influenced by the direction of Langer’s lines, orientation of collagen, and elastic fibers as well^52^^–^ 54. It was also observed on intact skin parts that vertical and horizontal stretching resulted in different tensile strength values. Results can be connected to clinically relevant risks for complications (*e.g.*, flap necrosis, dehiscence, thrombosis)^5^^,^ 17 ^,^ 44.

Limitations of the study include the number of experimental animals, the duration of the follow-up period, the selected ischemic time, general influencing factors of the wound healing. The follow-up period was only of two weeks. Accordingly, the inflammatory phase and the early days of the granulation phase of the wound healing process could be investigated. The ischemic time was arbitrarily chosen based on previous experiences^27^^–^ 29. Number of stitches and the suture material were also determined in the study. It is supposed that other suture materials and lower or higher number of stitches, as well as different suture types, would also influence the results.

As we plan to investigate the effects of various agents affecting wound healing and tissue regeneration, as an inert carrier, HPMC gel was used. In future studies, various agents will be able to be dissolved in the HPMC gel to study further the regeneration of flaps.

## Conclusion

The regeneration of the flaps was well monitored during the experiment. The hematological and micro-rheological parameters reflected the acute phase reactions, showing red blood cell deformability impairment and enhanced aggregation in the early p.o. days. The microcirculatory values of the ischemic flaps were lower than the contralateral ones even two weeks after surgery. The Cytocam-IDF method was suitable for intra- and postoperative monitoring of flap viability. Macroscopically, the ischemic-side flaps shrunk to a greater extent. Histology revealed that the quantity of mastocytes was reduced, and the quantity and organization of collagen fibers were altered in ischemic flaps. The model seems to be suitable for further studies using longer follow-up period and various agent influencing the regeneration and wound healing.

## Data Availability

The data will be available upon request.

## References

[1] Nanda D, Sahu SA, Karki D, Kumar S, Mandal A (2018). Adipofascial perforator flaps: Its role in reconstruction of soft-tissue defects of lower leg and ankle. Indian J Plast Surg.

[2] Kim KJ, Ahn JT, Yoon KT, Lee JH (2019). A comparison of fasciocutaneous and adipofascial methods in the reverse sural artery flap for treatment of diabetic infected lateral malleolar bursitis. J Orthop Surg (Hong Kong).

[3] Mégevand V, Suva D, Mohamad M, Hannouche D, Kalbermatten DF, Oranges CM (2022). Muscle vs. fasciocutaneous microvascular free flaps for lower limb reconstruction: A meta-analysis of comparative studies. J Clin Med.

[4] Raja BS, Vathulya M, Maheshwari V, Gowda AKS, Jain A, Kandwal P (2022). No added benefits of adipofascial flaps over fasciocutaneous flaps except for footwear ease and bulkiness: A systematic review and meta-analysis. J Clin Orthop Trauma.

[5] Yin X, Feng L, Hua Q, Ye J, Cai L (2024). Progress in the study of mechanisms and pathways related to the survival of random skin flaps. Updates Surg.

[6] Cokelet GR, Meiselman HJ, Baskurt OK, Hardeman MR, Rampling MW, Meiselman HJ (2007). Handbook of hemorheology and hemodynamics.

[7] Baskurt OK, Baskurt OK, Hardeman MR, Rampling MW, Meiselman HJ (2007). Handbook of hemorheology and hemodynamics.

[8] Pickett JA, Thorniley MS, Carver N, Jones DP (2003). Free flap monitoring in plastic and reconstructive surgery. Adv Exp Med Biol.

[9] Meier JK, Prantl L, Müller S, Moralis A, Liebsch G, Gosau M (2012). Simple, fast and reliable perfusion monitoring of microvascular flaps. Clin Hemorheol Microcirc.

[10] Meier JK, Prantl L, Geis S, Mueller S, Hullmann M, Liebsch G, Gosau M (2013). Luminescence ratiometric oxygen imaging (LROI) in microvascular anastomosed fibular and radial forearm flaps. Clin Hemorheol Microcirc.

[11] Yousefi S, Qin J, Dziennis S, Wang RK (2014). Assessment of microcirculation dynamics during cutaneous wound healing phases in vivo using optical microangiography. J Biomed Opt.

[12] Halani SH, Hembd AS, Li X, Kirby B, Beard CC, Haddock NT, Suszynski TM (2020). Flap monitoring using transcutaneous oxygen or carbon dioxide measurements. J Hand Microsurg.

[13] Mücke T, Hapfelmeier A, Schmidt LH, Fichter AM, Kanatas A, Wolff KD, Ritschl LM (2020). A comparative analysis using flowmeter, laser-Doppler |spectrophotometry, and indocyanine green-videoangiography for detection of vascular stenosis in free flaps. Sci Rep.

[14] Becker P, Blatt S, Pabst A, Heimes D, Al-Nawas B, Kämmerer PW, Thiem DGE (2022). Comparison of hyperspectral imaging and microvascular Doppler for perfusion monitoring of free flaps in an in vivo rodent model. J Clin Med.

[15] Ogura Y, Okamura M, Kataoka Y (2023). Rethinking perfusion assessment techniques in free flap reconstructive surgery. J Plast Reconstr Aesthet Surg.

[16] Ooms M, Heitzer M, Winnand P, Bock A, Katz M, Bickenbach J, Hölzle F, Modabber A (2023). Impacts of vascular comorbidities on free flap perfusion in microvascular head and neck reconstruction. Eur Arch Otorhinolaryngol.

[17] van den Heuvel MG, Buurman WA, Bast A, van der Hulst RR (2009). Review: Ischaemia-reperfusion injury in flap surgery. J Plast Reconstr Aesthet Surg.

[18] Martin RF (2020). Wound healing. Surg Clin North Am.

[19] Sorg H, Sorg CGG (2023). Skin wound healing: Of players, patterns, and processes. Eur Surg Res.

[20] Zhang F, Sones WD, Lineaweaver WC (2001). Microsurgical flap models in the rat. J Reconstr Microsurg.

[21] Casal D, Pais D, Iria I, Mota-Silva E, Almeida MA, Alves S, Pen C, Farinho A, Mascarenhas-Lemos L, Ferreira-Silva J, Ferraz-Oliveira M, Vassilenko V, Videira PA, Gory O’Neill J (2017). A model of free tissue transfer: The rat epigastric free flap. J Vis Exp.

[22] Hsu CE, Shyu VB, Wen CJ, Wei FC, Huang XT, Cheng HY (2018). The rat groin flap model redesigned for evaluating treatment effects on ischemia-reperfusion injury. J Surg Res.

[23] Ballestín A, Casado JG, Abellán E, Vela FJ, Álvarez V, Usón A, López E, Marinaro F, Blázquez R, Sánchez-Margallo FM (2018). Ischemia-reperfusion injury in a rat microvascular skin free flap model: A histological, genetic, and blood flow study. PLoS One.

[24] Ballestín A, Casado JG, Abellán E, Vela FJ, Campos JL, Martínez-Chacón G, Bote J, Blázquez R, Sánchez-Margallo FM (2019). A pre-clinical rat model for the study of ischemia-reperfusion injury in reconstructive microsurgery. J Vis Exp.

[25] Aksamitiene E, Heffelfinger RN, Hoek JB, Pribitkin ED (2024). Standardized pre-clinical surgical animal model protocol to investigate the cellular and molecular mechanisms of ischemic flap healing. Biol Proced Online.

[26] Tamas R, Nemeth N, Brath E, Sasvari M, Nyakas C, Debreczeni B, Miko I, Furka I (2010). Hemorheological, morphological, and oxidative changes during ischemia-reperfusion of latissimus dorsi muscle flaps in a canine model. Microsurgery.

[27] Klarik Z, Tamas R, Toth E, Kiss F, Kovacs EL, Jäckel M, Furka I, Nemeth N (2015). Intra and postoperative evaluations of microcirculation and micro-rheological parameters in a rat model of musculocutaneous flap ischemia-reperfusion. Acta Cir Bras.

[28] Molnar A, Magyar Z, Nachmias DB, Mann D, Szabo B, Toth L, Nemeth N (2020). Effect of short-term ischemia on microcirculation and wound healing of adipocutaneous flaps in the rat. Acta Cir Bras.

[29] agyar Z, Molnar A, Nachmias DB, Mann D, Sogor V, Mester A, Peto K, Nemeth N (2021). Impact of groin flap ischemia-reperfusion on red blood cell micro-rheological parameters in a follow-up study on rats. Clin Hemorheol Microcirc.

[30] Green CJ, Knight J, Precious S, Simpkin S (1981). Ketamine alone and combined with diazepam or xylazine in laboratory animals: a 10 year experience. Lab Anim.

[31] Flecknell P (2015). Laboratory animal anaesthesia.

[32] Cannon CZ, Kissling GE, Hoenerhoff MJ, King-Herbert AP, Blankenship-Paris T (2010). Evaluation of dosages and routes of administration of tramadol analgesia in rats using hot-plate and tail-flick tests. Lab Anim (NY).

[33] Lundell A, Bergqvist D, Mattsson E, Nilsson B (1993). Volume blood flow measurements with a transit time flowmeter: an in vivo and in vitro variability and validation study. Clin Physiol.

[34] Mordick TG 2nd, Romanowski L, Eaton C, Siemionow M (1995). Microvascular application of the nonreversed vein graft. Plast Reconstr Surg.

[35] Aykut G, Veenstra G, Scorcella C, Ince C, Boerma C (2015). Cytocam-IDF (incident dark field illumination) imaging for bedside monitoring of the microcirculation. Intensive Care Med Exp.

[36] Hutchings S, Watts S, Kirkman E (2016). The Cytocam video microscope. A new method for visualising the microcirculation using Incident Dark Field technology. Clin Hemorheol Microcirc.

[37] De Backer D, Hollenberg S, Boerma C, Goedhart P, Büchele G, Ospina-Tascon G, Dobbe I, Ince C (2007). How to evaluate the microcirculation: report of a round table conference. Crit Care.

[38] Hardeman MR, Goedhart PT, Shin S, Baskurt OK, Hardeman MR, Rampling MW, Meiselman HJ (2007). Handbook of hemorheology and hemodynamics.

[39] Baskurt OK, Boynard M, Cokelet GC, Connes P, Cooke BM, Forconi S, Liao F, Hardeman MR, Jung F, Meiselman HJ, Nash G, Nemeth N, Neu B, Sandhagen B, Shin S, Thurston G, Wautier JL, International Expert Panel for Standardization of Hemorheological Methods (2009). New guidelines for hemorheological laboratory techniques. Clin Hemorheol Microcirc.

[40] Baskurt OK, Hardeman MR, Uyuklu M, Ulker P, Cengiz M, Nemeth N, Shin S, Alexy T, Meiselman HJ (2009). Parameterization of red blood cell elongation index–shear stress curves obtained by ektacytometry. Scand J Clin Lab Invest.

[41] Fazekas LA, Szabo B, Szegeczki V, Filler C, Varga A, Godo ZA, Toth G, Reglodi D, Juhasz T, Nemeth N (2023). Impact assessment of pituitary adenylate cyclase activating polypeptide (PACAP) and hemostatic sponge on vascular anastomosis regeneration in rats. Int J Mol Sci.

[42] Godo ZA, Fazekas LA, Fritsch G, Szabo B, Nemeth N (2024). A custom-developed device for testing tensile strength and elasticity of vascular and intestinal tissue samples for anastomosis regeneration research. Sensors (Basel).

[43] Rohrich RJ, Weinstein A (2015). The impact of plastic and reconstructive surgery: by the numbers. Plast Reconstr Surg.

[44] Phillips BT, Lanier ST, Conkling N, Wang ED, Dagum AB, Ganz JC, Khan SU, Bui DT (2012). Intraoperative perfusion techniques can accurately predict mastectomy skin flap necrosis in breast reconstruction: results of a prospective trial. Plast Reconstr Surg.

[45] Berthelot M, Yang GZ, Lo B (2018). A self-calibrated tissue viability sensor for free flap monitoring. IEEE J Biomed Health Inform.

[46] Jeon FHK, Varghese J, Griffin M, Butler PE, Ghosh D, Mosahebi A (2018). Systematic review of methodologies used to assess mastectomy flap viability. BJS Open.

[47] Khavanin N, Qiu C, Darrach H, Kraenzlin F, Kokosis G, Han T, Sacks JM (2019). Intraoperative perfusion assessment in mastectomy skin flaps: How close are we to preventing complications?. J Reconstr Microsurg.

[48] Patial T, Kaur A, Mittal R (2022). Use of intra operative fluorescein dye for prediction of flap viability. Pol Przegl Chir.

[49] Zhou Z, Yu L, Meng F, Wen J, Xiao Y, Wan S, Zeng H, Yu F (2024). Study on blood circulation monitoring after the tissue transfer of ultrathin or conventional anterolateral thigh flaps. Plast Reconstr Surg Glob Open.

[50] Kim HH, Song IS, Cha RJ (2024). Advancing DIEP flap monitoring with optical imaging techniques: A narrative review. Sensors (Basel).

[51] Akhavani MA, Sivakumar B, Paleolog EM, Kang N (2008). Angiogenesis and plastic surgery. J Plast Reconstr Aesthet Surg.

[52] Zellner EM, Hedlund CS, Kraus KH, Burton AF, Kieves NR (2016). Comparison of tensile strength among simple interrupted, cruciate, intradermal, and subdermal suture patterns for incision closure in ex vivo canine skin specimens. J Am Vet Med Assoc.

[53] Gökkaya A, Görgü M, Kızılkan J, Karanfil E, Doğan A (2022). The measurement of wound tensile strength and the effect of PRP on wound tensile force: an experimental investigation on rabbits. J Plast Surg Hand Surg.

[54] Capek L, Flynn C, Molitor M, Chong S, Henys P (2018). Graft orientation influences meshing ratio. Burns.

